# A Rare Evidence of a Dual Atrioventricular Nodal Physiology in a Patient with Narrow Complex Tachycardia

**DOI:** 10.19102/icrm.2018.091105

**Published:** 2018-11-15

**Authors:** Khalil Kanjwal

**Affiliations:** ^1^McLaren Cardiovascular Group, Michigan State University, Lansing, MI, USA

**Keywords:** Ablation, AVNRT, dual AV node, SVT

## Abstract

The present case details an interesting intracardiac electrogram in a patient who demonstrated recurrent episodes of narrow complex tachycardia and who was subsequently found to have a typical atrioventricular nodal reentrant tachycardia during an electrophysiology study. The patient subsequently underwent slow pathway ablation and was found to be noninducible for any tachycardia after ablation.

## Introduction

**Figure 1:**
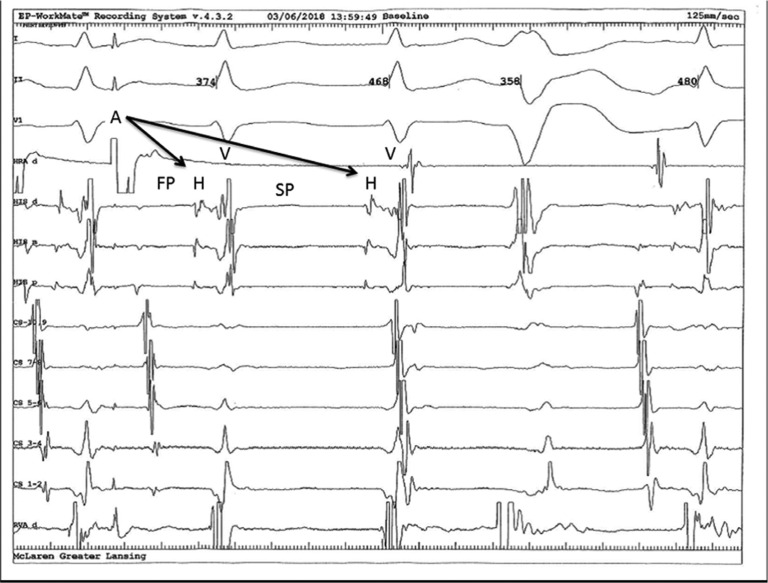
Intracardiac EGM showing the phenomenon of a double-fire with antegrade conduction down both the fast and slow AV nodal pathways. There is also an atrial echo noted on the second QRS of the double-fire.

Atrioventricular (AV) nodal reentry tachycardia (AVNRT) is the most common supraventricular tachycardia (SVT) in adults. Patients with AVNRT have a dual AV node consisting of slow and fast pathways. Various electrophysiological findings and maneuvers can prove the AVNRT to be a mechanism of tachycardia during electrophysiology study (EPS).^1,2^ The most commonly used definition of a dual AV nodal physiology is the jump and echo phenomenon noted during an EPS. Jump is defined as an increase in the A–H interval of more than 50 ms, with a 10 ms decrement in an extrastimuli or a 10 ms decrement in an atrial drive train.^2^ Rarely, patients can have simultaneous conduction down both the slow and fast pathways, resulting in two QRS complexes for a single P-wave. On the electrogram (EGM), this phenomenon is seen as an atrial EGM followed by two ventricular EGMs (ie, two–for-one phenomenon or a “double-fire”). In this case report, a tracing showing a two-for-one phenomenon (“double-fire”) in a patient who had recurrent palpitations and AVNRT is presented **(Figure 1)**.

## Case history

A 67-year-old male with a longstanding history of palpitations was seen in our arrhythmia clinic and was offered participation in an EPS. The patient was brought to the electrophysiology laboratory. After proper sedation and completion of the usual sterile drape and preparation protocols, electrophysiology catheters were advanced under fluoroscopy to the right ventricular apex, His region, high right atrium, and coronary sinus. EPS was performed and, during atrial extrastimuli, the findings noted in the tracing were consistently reproduced.

Notably, the patient had an extrastimulus followed by two R-wave EGMs. There was an echo beat observed on the second R-wave. The first R-wave resulted from the antegrade conduction over the fast pathway. The second R-wave resulted from the antegrade conduction over the slow pathway way. This phenomenon has been reported previously^3,4^ but is more commonly seen in females in the fifth decade of their life.

During EPS, an echo was noted with the retrograde conduction over the fast pathway. However, this phenomenon failed to initiate the tachycardia as the premature ventricular complex (PVC) came in. In the patient, the PVC was observed to find the AV node refractory (concealed conduction from retrograde conduction), as it had just been depolarized by an echo beat and conducted retrogradely with a long V–A interval over the slow pathway. Although this is a known phenomenon, what makes this tracing interesting is the presence of an atrial echo on the second QRS. An atrial echo seen on the second QRS is rare during a double-fire and initiation of AVNRT. There are reports of initiation of nonreentrant tachycardia by simultaneous conduction over fast and slow pathways, and these often lead to cardiomyopathies.^5–7^

The patient was subsequently induced for an SVT with a very short septal VA interval. Ventricular entrainment during tachycardia resulted in a VAHV response and a long postpacing interval suggestive of an AVNRT as a mechanism of the tachycardia. Slow pathway ablation was performed with complete elimination of any slow pathway conduction and the patient was noninducible for any tachycardia after the ablation.

It is possible that the next echo beat could have been a junctional ectopic. However, this finding was reproducible and the patient had inducible AVNRT and, after ablation, this finding could not be reproduced. Thus, it is believed that the second ventricular complex is because of simultaneous conduction over the slow pathway.
